# Parallel Recovery Dynamics of Circulating and Tissue-Resident Hemocytes Following Hemolymph Withdrawal in *Pomacea canaliculata*

**DOI:** 10.3390/ijms27156619

**Published:** 2026-07-24

**Authors:** Chiara Losi, Anita Ferri, Monica Montanari, Nicola Franchi, Gabriele Sansalone, Sandro Sacchi, Davide Malagoli

**Affiliations:** 1 Department of Life Sciences, University of Modena and Reggio Emilia, 41125 Modena, Italy; chiara.losi@unimore.it (C.L.); anitaferri@unimore.it (A.F.); monica.montanari@unimore.it (M.M.); nicola.franchi@unimore.it (N.F.); gabriele.sansalone@unimore.it (G.S.); sandro.sacchi@unimore.it (S.S.); 2 Department of Chemical and Geological Sciences, University of Modena and Reggio Emilia, 41125 Modena, Italy; 3 NBFC—National Biodiversity Future Center, 90133 Palermo, Italy

**Keywords:** apple snail, gastropod, eco-immunology, FISH, hematopoiesis, hemocyte reservoir, histology, image analysis, immunity, immunocyte

## Abstract

In vertebrates, circulating and tissue-resident immune cells contribute to host defense and tissue homeostasis. Their balance is maintained through hematopoiesis and anatomical reservoirs. In mollusks, this balancing is poorly investigated. Thus, the dynamics of circulating and tissue-resident hemocytes after a hemolymph withdrawal were analyzed in *Pomacea canaliculata*, a snail that allows repeated hemolymph withdrawals and possesses posterior kidney (PK) cell aggregates known as hemocyte islets. In the absence of specific hemocyte markers, flow cytometry data revealed dynamic changes in circulating hemocyte number and populations within 48 h following hemolymph withdrawal. In addition, computer-assisted image analysis of histological sections showed a significant transient reduction in PK hemocyte-islet area. Analyses of publicly available *P. canaliculata* transcriptomes revealed predominant and highly variable expression of two hematopoiesis-related genes, namely Transglutaminase (*Pc*TGase) and Hematopoietically expressed homeobox protein (*Pc*Hhex), in circulating hemocytes and PK. Fluorescence in situ hybridization highlighted constitutive *Pc*TGase and *Pc*Hhex expression within PK hemocyte islets. RT-qPCR experiments confirmed transcriptomic data on *Pc*TGase and *Pc*Hhex constitutive expression, without significant modulation following hemolymph withdrawal. Collectively, these findings provide the first evidence of parallel recovery dynamics involving circulating hemocytes and PK hemocyte islets in *P. canaliculata*.

## 1. Introduction

The immune system can be defined as the repository of the body’s capacity to dynamically recognize between self- and non-self-elements and activate context-dependent responses to tolerate or counteract the latter [1]. In this view, switching from the defensive/aggressive role to a more physiological perspective, the immune system can be considered also acting as an important player in maintaining the homeostasis of the organism [2]. Invertebrates’ immune system, which is innate only, relies on both cellular and humoral components [3]. As immune competent cells are typically retrieved circulating in the hemolymph, they are usually referred to as either hemocytes or immunocytes [4]. Hemocytes have traditionally been classified according to morphological features, including cell size, shape, and cytoplasmic granularity; however, a unified nomenclature has not been established [5]. Recent studies have shown that similar morphologies can correspond to distinct molecular profiles, pointing to a higher level of specialization and a broader functional repertoire than previously appreciated [6].

In both vertebrates and invertebrates, the cellular component of the immune system is sustained throughout life by differentiation mechanisms from hematopoietic stem cells (HSCs) through the process of hematopoiesis. HSC proliferation often takes place in specialized hematopoietic organs that provide specific micro-environments, i.e., hematopoietic niches, where undifferentiated stem cells can self-renew and produce progeny that differentiate into different blood cell types [7]. While immunology and hematopoiesis are fields of intensive research in vertebrates and a few of non-vertebrate organisms that serve as developmental and immunological models, e.g., *Drosophila melanogaster* [8], the origin of hemocytes and the mechanisms maintaining equilibrium between circulating and tissue-resident hemocytes in most invertebrate phyla remain poorly studied and not yet understood [9,10].

Mollusks are a large phylum that includes several long-lived organisms, many of which are known for their economic and biomedical importance [11]. Within this group, both the hemocyte functional specialization and the localization of hematopoietic sites vary considerably [11]. In the oyster *Magallana gigas* (*Crassostrea gigas*), a population of adult somatic progenitors has been identified in the gills that give rise to differentiated hemocytes [12], whereas mitotic spindles in circulating hemocytes have been reported in the clam *Tapes philippinarum* [13]. Single-cell analysis of circulating hemocytes from *M. gigas* identified multiple distinct transcriptomic clusters with potential lineage relationships, suggesting functional specialization, but canonical stem or progenitor cell populations were not clearly resolved [14]. Recently, an HSC niche was proposed in adult bivalve shells, resembling vertebrate bone marrow [15], opening new perspectives for hemocyte origin studies in mollusk models. In gastropods, hematopoietic events have been documented close to the reno-pericardial zone [16]. The presence of a specific hematopoietic site within this region, named the amebocyte-producing organ (APO), has been described in *Biomphalaria glabrata* and other snails [16,17]. However, its central role as hematopoietic organ has been debated [18], and a multicentric origin for the hemocytes has also been proposed. Single-cell RNA-seq experiments revealed high heterogeneity and plasticity within *B. glabrata* circulating hemocytes, whereas cell lineage relationships could not be definitively established [19]. In all, questions regarding hemocyte origin, turnover, population balancing, and functional specialization are still open in mollusks.

The freshwater snail *Pomacea canaliculata* represents a particularly suitable model to investigate hemocyte origin and turnover dynamics. This species offers the unique possibility to perform repeated hemolymph withdrawals without affecting survival, thereby enabling investigation of hemocyte replenishment and trafficking between circulation and tissue compartments [20]. Beyond comparative immunology [21,22,23], *P. canaliculata* is increasingly used as a model across multiple fields, including ecotoxicology [24,25,26,27,28], parasitology [29,30], neuroimmunology [31,32], and regeneration studies [33,34,35,36,37]. Its high environmental resilience [38,39], invasiveness [21], and expanding omics and functional toolkits [40,41,42,43,44,45] further strengthen its value for studying hemocyte origin and dynamics. *P. canaliculata* circulating hemocytes have been categorized as either small, blast-like Group I (GI) cells or large, agranular or granular Group II (GII) cells, with phagocytic activity restricted to a subset of cells within GII [46,47]. Although a circulating hemocyte-specific proteome has been released [48] and a small subpopulation of circulating hemocytes has been hypothesized to retain proliferative or progenitor potential [49], reliable markers enabling the tracking of hemocyte differentiation or lineages have not yet been identified [47]. Similar to other gastropods, immunohistochemical evidence suggested that *P. canaliculata* hematopoiesis may occur within the pericardial cavity [20]. In addition, several organs, including posterior kidney (PK), lung, and gills, have been suggested as hematopoietic sites or hemocyte-containing organs, as well as immune barriers [22,50,51,52]. Specifically, tissue-resident phagocytic cells have been found in PK, forming aggregates named hemocyte islets, in which phagocytosis has been shown to occur after injection of microbial suspension [50]. Although the hemocyte islets have been proposed as hematopoietic sites [51], their contribution to physiological hemocyte turnover, as well as their relationship with circulating hemocyte dynamics, remains unresolved. In this context, the existence of hemocyte reservoirs that may buffer hemocyte loss following bleeding remains unexplored in gastropods. In *P. canaliculata* repeated hemolymph withdrawals at 24 h intervals had an impact on ampulla morphology but did not significantly alter the balance between GI and GII hemocyte populations, suggesting the existence of control mechanisms over the proportion of circulating cells [20]. Similar observations came from experiments on *Lymnaea stagnalis* [53]. This steady balance may be fundamental given the specific yet undisclosed functions that hemocytes perform within and outside the circulation.

Given the limited information on hemocyte balance and the involvement of hemocyte-containing organs in maintaining circulating cell homeostasis, this study investigated hemolymph repopulation and the response of the PK following hemolymph withdrawal in *P. canaliculata*. First, we assessed the temporal dynamics of circulating hemocyte number and populations within 48 h following the first hemolymph withdrawal, through acoustic flow cytometry complemented with a paired-sample statistical analysis and a model-based temporal series approach. In parallel, we explored the effects of hemolymph withdrawal on the PK using histological and molecular approaches, to determine whether changes occur simultaneously in circulating hemocyte populations and hemocyte islets. In the absence of recognized hemocyte markers [47], we selected *Pc*-Transglutaminase (*Pc*TGase) and *Pc*-Hematopoietically expressed homeobox protein (*Pc*Hhex), two molecules associated with clotting and hematopoiesis in both vertebrates and invertebrates [54,55], as proxies for hemocyte presence. Their expression in circulating cells and PK was first analyzed in silico using available transcriptomic datasets [32]. Fluorescence in situ hybridization (FISH) was then employed to assess whether *Pc*TGase and *Pc*Hhex expression in the PK was associated with hemocyte islets. Finally, their expression was examined by quantitative PCR (RT-qPCR) in the PK at specific time points following hemolymph withdrawal. Overall, the collected data indicate that the PK responds to hemocyte loss through transient changes in hemocyte islets and reveal parallel recovery dynamics in the hemolymph and PK, raising the possibility of coordinated balance between circulating hemocytes and tissue-resident hemocyte islets in *P. canaliculata*.

## 2. Results

### 2.1. Transient Changes in Circulating Hemocyte Populations Consistent with Non-Directional Temporal Dynamics Following Hemolymph Withdrawal

Acoustic flow cytometry analysis was performed to assess circulating hemocyte composition and abundance during the hemolymph repopulation process. Each animal was subjected to two hemolymph withdrawals: a first (time 0) and a second at 1.5, 3, 6, 9, 18, 24, or 48 h post-withdrawal (hpw). Two gates were defined on the dot plots to distinguish between the two hemocyte groups (i.e., small GI-like and large GII-like). The two gates were consistently applied across all samples. The ratio of GI-like to total number of gated hemocytes (GI + GII) and the total number of gated hemocytes were considered as parameters to assess hemolymph recovery. GI-like hemocytes increased significantly after 6, 9, and 18 hpw (Figure 1A). The total number of gated hemocytes increased significantly after 24 hpw (Figure 1B). No significant differences between first and second withdrawals were detected at the remaining time points, including 48 hpw. To describe the overall temporal dynamics of hemocyte populations, we complemented the paired analysis with a model-based time-series analysis. Four alternative evolutionary models were fitted: Stasis, Unbiased Random Walk (URW), General Random Walk (GRW), and Ornstein–Uhlenbeck (OU). Collected data indicate that variation in the proportion of GI hemocytes over time was most strongly supported by the URW model, with weaker support for alternative models (Figure 2A). Similarly, for the total number of gated hemocytes, the temporal variation was best explained by URW, with additional support for the Stasis model (Figure 2B). These temporal profiles were consistent with the absence of significant differences between the first and second withdrawals at 48 hpw.

### 2.2. Transient Reduction in PK A_hi_/A_tot_ Ratio in Absence of Cyto-Histological Alterations Following Hemolymph Withdrawal

To morphologically assess the response of PK hemocyte islets to hemolymph withdrawal, histological analysis was performed on PKs collected at 1.5, 6, 18, 24, and 48 hpw, along with controls. The area occupied by hemocyte islets (A_hi_) relative to the section area (A_tot_) was calculated as the A_hi_/A_tot_ ratio (Figure 3A). The A_hi_/A_tot_ ratio was significantly reduced at 18 and 24 hpw compared to controls, whereas no significant differences were detected at 1.5, 6, or 48 hpw (Figure 3B). Notably, hematoxylin–eosin-stained sections showed no structural differences in the renal hemocyte islets, still intact within the crypts, or in the surrounding parenchyma at any experimental time point (Figure 4). Subsequently, to characterize the overall temporal pattern of change in hemocyte islet area, a model-based time-series analysis was applied as previously described. Model comparison indicated that the temporal trend within 48 hpw was best explained by an URW, with additional support for a Stasis model. Models implying directional change received substantially lower support (Figure S1), which is consistent with the absence of significant differences between the 48 hpw time point and the control conditions.

Additionally, a morphological analysis of circulating hemocytes was performed following staining, with a focus on nuclear morphology to assess the presence of mitotic figures during the hemolymph repopulation. No mitotic cells were detected in either control (0 hpw) or 9 hpw samples (Figure S2).

### 2.3. PcTGase and PcHhex Expression in Circulating Hemocytes and PK Hemocyte Islets

The genes *Pc*TGase (Gene ID: LOC112576690) and *Pc*Hhex (Gene ID: LOC112564124) were selected based on their known involvement in clotting and hematopoietic processes in both vertebrates and invertebrates [54,55]. Conserved domain analysis performed using SMART [56] was consistent with a conserved functional role for these proteins in *P. canaliculata* (Supplementary Figure S3). Their expression was initially investigated using transcriptomic datasets available in the NCBI Sequence Read Archive (SRA), considering 134 tissue/organ-specific transcriptomes representing tissues with more than three biological replicates (Supplementary Table S2). Transcriptomic data analysis revealed that circulating hemocytes and PK exhibited the highest average expression levels of both *Pc*TGase (LOC112576690, Figure 5A) and *Pc*Hhex (LOC112564124, Figure 5B), compared to other analyzed tissues.

To evaluate whether the expression highlighted in PK by the transcriptomic analysis was due to a specific cellular component, FISH experiments were performed on control PKs. Constitutive *Pc*TGase and *Pc*Hhex expression was observed predominantly within hemocyte islets (Figure 6).

### 2.4. Conserved Expression of PcTGase and PcHhex in PK Following Hemolymph Withdrawal

The expression of *Pc*TGase and *Pc*Hhex was assessed by RT-qPCR in PK samples collected at 18 and 24 hpw (Figure 7). Compared with control (i.e., no hemolymph withdrawal), neither gene showed significant differences at either time point.

## 3. Discussion

In invertebrates, the immune cellular component is represented by the hemocytes [5], also defined as immunocytes [4], which play essential roles in pathogen defense, development, and tissue homeostasis [9,57,58]. Hemocytes are present both as circulating cells in the hemolymph and as resident cells within tissues, and their physiological balance relies on hematopoiesis and exchanges between tissue reservoirs and circulation [5]. The existence of mechanisms and organs involved in the control of hemocyte balance remain largely unexplored in mollusks [11]. In this field, the laboratory-bred freshwater snail *P. canaliculata* is a valuable research model for investigating hemocyte balancing, thanks to the possibility to perform multiple hemolymph withdrawals from the same animal [20] and the availability of omics data [32,40,41]. As it is common for invertebrate models, *P. canaliculata* circulating hemocyte populations have been first categorized on the basis of their size and cytoplasmic granularity [46,50]. Advanced label-free and image-based flow cytometry subsequently identified seven hemocyte clusters [47], that can be largely associated with the hemocyte morphotypes previously categorized into Group I, i.e., cells of small or intermediate size and agranular cytoplasm, and Group II, i.e., large cells presenting either agranular or granular cytoplasm [46]. Tissue-resident hemocytes have been reported in several organs of *P. canaliculata*, primarily the posterior kidney [50,51,52]; however, the relationship between hemolymph repopulation dynamics and tissue-resident hemocytes has not been explored yet.

To investigate the dynamics of circulating and PK-resident hemocytes after hemolymph withdrawal in *P. canaliculata*, we performed paired hemolymph withdrawals with the second withdrawal after experimental time points extending from 1.5 to 48 hpw. Additionally, we evaluated cell aggregate (i.e., hemocyte islets [51]) area as the A_hi_/A_tot_ ratio on PKs collected after hemolymph withdrawal. We first examined time-specific variations and subsequently assessed global temporal dynamics [59,60,61,62,63]. While a minor amount of hemolymph (<2 mL) could be recovered in the early time points (1.5 and 3 hpw), no significant differences in either morphotype composition or hemocyte number were observed with paired statistical analyses of flow cytometry data. However, after 6 hpw, the proportion of circulating GI hemocytes increased, reaching a maximum at 18 hpw, suggesting the presence of mechanisms aimed at compensating for hemocyte loss. During this phase, the increase in small GI hemocytes into the circulation could be due either to their release from reservoir organs or to their de novo production in hematopoietic sites [20,51]. At 18 hpw, the total number of gated hemocytes rose, peaking at 24 hpw, when the GI/GII ratio did not differ significantly from the initial condition. At 48 hpw, hemolymph samples presented no significant differences from those collected at time 0. Consistently, model-based time-series analysis indicated that the dynamics of hemolymph repopulation are best described by the Unbiased Random Walk model, which describes successive changes that lack a consistent directional trend. These hemocyte dynamics may either rely on the early recruitment of small hemocytes into the circulation and their rapid maturation into larger GII-like hemocytes, or it could depend on the release in circulation of GI and GII hemocytes with different timing. Also, at this stage, the coexistence of both processes cannot be excluded. Despite intensive studies, there is still no consensus about the mechanisms that govern hemocyte proliferation and differentiation in gastropods [11]. Consequently, whether the two hemocyte groups represent different stages of the maturation of a single hemocyte lineage or of two different cell lineages remains unresolved, and even in-depth flow cytometric analyses have not provided conclusive evidence [47]. Several authors indicated a common hematopoietic center located in the reno-pericardial region of gastropods [16,17], while other authors suggested that the origin of hemocytes is polycentric [18] or might occur in circulation [64]. No occurrence of physiological mitosis has been documented in the hemolymph of *P. canaliculata* so far [20,47,49], though a small proportion of circulating and potentially proliferating hemocytes has been reported [49]. The present microscopic analysis of circulating hemocytes performed at 9 hpw, i.e., before the peak of GI-like hemocytes, did not reveal active mitosis among circulating hemocytes during hemolymph repopulation, consistent with previous observations following hemolymph withdrawal [20]. While not excluding that some circulating hemocytes can present a proliferative capacity [49,64], collected data prompted us to investigate the mobilization of hemocytes from hemocyte-containing organs as a possible mechanism contributing to hemolymph repopulation following significant bleeding, rather than proliferation of circulating hemocytes in the absence of tissue damage or other specific trauma.

In *P. canaliculata*, it has been hypothesized that the PK may act as a hematopoietic site, in addition to functioning as a hemocyte-containing organ and immune barrier [50,51]. The PK lies beneath the mantle and is formed by numerous epithelial crypts consisting of a cylindrical epithelium, while in the interstices there are aggregates of cells, labeled as hemocyte islets [50,51], but no information is available regarding the relationship between PK-resident and circulating hemocyte dynamics. In the absence of established hemocyte markers and cell-tracing resources in *P. canaliculata*, the discrete organization of hemocyte islets currently makes the PK the most suitable organ for the quantitative assessment of changes in tissue-resident hemocyte-like cells by histological analysis. Histological analysis performed on PKs following hemolymph withdrawal did not find evidence of structural damages in hemocyte islets or epithelial crypts in any of the analyzed time points. Computer-assisted image analysis showed a significant decrease in hemocyte islet areas (A_hi_/A_tot_) at 18 hpw, concomitantly with the peak for GI hemocytes observed by flow cytometry. At 24 hpw, A_hi_/A_tot_ is still significantly smaller, though recovering. Consistently, model-based time-series analysis of A_hi_/A_tot_ suggested a progressive recovery at 48 hpw, when circulating hemocyte parameters were not significantly different from baseline. Overall, these observations corroborate the hypothesis that *P. canaliculata* can finely regulate the number and the proportions of circulating hemocyte morphotypes [20]. In the absence of known hemocyte markers that could allow for hemolymph repopulation and hemocyte islet recovery in vivo, our time-based flow cytometry and computer-assisted image analysis suggest that PK may be involved in hemolymph repopulation after hemocyte loss. Present data are consistent with the possibility that PK hemocyte islets participate in the response to hemocyte loss, but the temporal mismatch between the GI increase, starting at 6 hpw, and the first significant shrinkage of hemocyte islets observed at 18 hpw suggests that other contributions including recruitment from other organs (e.g., gills and lung) or de novo hematopoiesis may be possible.

Based on these findings, the relationship between circulating hemocyte dynamics and PK cell aggregates was investigated at the molecular level. Looking for hematopoietically related transcripts, we first singled out *Pc*TGase and *Pc*Hhex transcripts based on conserved-domain analysis. We then analyzed 134 tissue/organ-specific publicly available *Pc* transcriptomes and observed that *Pc*TGase and *Pc*Hhex transcripts were predominantly expressed in both hemocytes and PK, with a high variability in the expression levels among replicates. On this basis, they were selected as potential proxies for hemocyte presence. The transglutaminase family encloses multifunctional enzymes, and it is an important component of the blood coagulation cascade [65]. Among invertebrates, a transglutaminase family component has been discovered in the limulus *Tachypleus tridentatus* [66] and its activity has been widely studied in the hemolymph coagulation cascade of crustaceans [67]. While circulating hemocytes exhibit transglutaminase activity in the crayfish *Pacifastacus leniusculus* [67], this enzyme has also been abundantly retrieved in the hematopoietic tissue cells, preventing hematopoietic stem cells from starting to differentiate and migrate into the hemolymph [54]. Inhibition of TGase activity loosens the extracellular matrix in the hematopoietic tissue, leading to the release of progenitor cells into the circulation [68,69]. Studies on the oyster *M. gigas* have shown that TGase can act as an immune-related coagulant protein [70]. Hhex is a key transcription factor in vertebrate hematopoiesis [55]. Its role has mainly been studied in mice, where it is essential for embryonic liver development and it is necessary for the maturation and proliferation of the earliest definitive hematopoietic progenitors [71,72], as well as for the maintenance of normal stem and progenitor cell populations in adult [73]. Orthologs have been found in *Caenorhabditis elegans* and in *D. melanogaster*, exhibiting non-hematopoietic functions [74,75]. Hhex has been shown to be highly expressed in the veliger larvae of *Mytilus galloprovincialis* and, among adult tissues, overexpressed in hemocytes [76], but no information is provided on its functions in mollusks. Given the predominant expression of *Pc*TGase and *Pc*Hhex in circulating hemocytes and the PK, FISH experiments were performed to determine whether their expression in the PK was associated with a specific cellular component. FISH revealed that *Pc*TGase and *Pc*Hhex transcripts were localized to cells within the hemocyte islets. Although *Pc*TGase and *Pc*Hhex cannot be considered exclusive hemocyte markers, these findings provide independent molecular support for the hemocyte-rich nature of PK islets.

RT-qPCR on PKs revealed variable expression levels for both *Pc*TGase and *Pc*Hhex in the control and after hemolymph withdrawals, confirming the high variability of their expression highlighted by transcriptomic analysis. Although the marked variability observed among biological replicates may mask transcriptional changes occurring in PK during the recovery process, RT-qPCR only confirmed *Pc*TGase and *Pc*Hhex constitutive expression, but not a significant change during renal islet depletion and recovery after hemolymph withdrawal. Future experiments are needed to establish the biological functions of *Pc*TGase and *Pc*Hhex in circulating hemocytes and PK.

In conclusion, the snail restored its hemolymph cellular composition through transient changes in hemocyte proportions, paralleled by a temporary reduction in PK hemocyte islets. *Pc*TGase and *Pc*Hhex showed constitutive expression in both circulating hemocytes and the PK, supporting the hemocyte-rich nature of the islets. On the whole, these results provide the first evidence of parallel recovery dynamics of circulating hemocytes and PK hemocyte islets in *P. canaliculata*, suggesting the existence of a balance between circulating and tissue-resident hemocyte compartments. These data indicate that *P. canaliculata* relies on mechanisms that involve extracirculatory compartments and regulate hemocyte levels, allowing the snail to cope with bleeding.

## 4. Materials and Methods

### 4.1. Animal Maintenance

The *Pomacea canaliculata* specimens used in this study are bred in the aquarium facility of the Department of Life Sciences, University of Modena and Reggio Emilia as described by Ferri et al. [32]. Animals are kept in aerated static aquaria at 25 ± 1 °C, light–dark photoperiod of 12:12 h, with a maximum density of 1 adult animal per 2 L of water. Routine maintenance, performed twice a week, includes partial water changes (approximately 80% of the water) and cleaning of tank surfaces. Following each water change, snails are fed fresh green salad. Only adult individuals (12–18 months old), including both males and females, were used in the experiments.

### 4.2. Hemolymph Withdrawal

Hemolymph withdrawal was performed by applying a gentle and continuous pressure onto the operculum of the snail [46], which is induced to retreat into its shell, releasing hemolymph which can be easily identified by its bluish color. Snails were starved for 3 days before the hemolymph withdrawal to obtain samples as clean as possible. For each animal, the maximum amount of hemolymph released at each withdrawal (1–6 mL, depending on the size of individual *P. canaliculata* specimens) was collected, and no mortality was observed in consequence of the withdrawal procedure. Hemolymphs were kept separated and never pooled.

### 4.3. Flow Cytometry and Statistical Analysis of Hemocyte Dynamics

Hemolymph samples from 84 snails were analyzed by flow cytometry using a paired experimental design. Each animal underwent an initial hemolymph withdrawal (time 0), followed by a second withdrawal after 1.5, 3, 6, 9, 18, 24, or 48 h post-withdrawal (hpw), with 12 animals analyzed at each time point. Hemocyte repopulation was therefore assessed within the same individual by comparing the two paired hemolymph samples. Attune™ NxT^®^ Acoustic Focusing Cytometer (ThermoFisher Scientific, Waltham, MA, USA) was employed for this analysis, which applies an ultrasound-based acoustic technology that allows for a very fast analysis of circulating hemocytes. To distinguish single cells from aggregates, a doublet discriminator comparing FSC-H vs. FSC-A parameters was applied [36]. Additionally, CellTracker™ Green CMFDA (ThermoFisher Scientific, Waltham, MA, USA), a vital stain that requires no cell permeabilization, was employed to distinguish hemocytes from debris. A 10 mM CMFDA stock solution in DMSO was diluted 1:100 to prepare a 100 μM working solution in filtered Freshwater Snail Solution (FSS: 41.15 mM NaCl, 0.54 mM KCl, 3.55 mM CaCl_2_, 2.61 mM MgCl_2_, 5 mM Tris, pH 7.5) [77] and used at a final concentration of 1 μM per sample. Samples were incubated 15 min on ice in the dark before analysis. The number of events was fixed at 40,000 for each sample [36]. Two gates corresponding approximately to the main hemocyte morphotypes (GI and GII [46]) were defined into the dot plot based on FSC and SSC parameters and consistently applied to all the paired samples at time 0 and subsequent time points.

As two hemolymph withdrawals were performed for each individual (i.e., an initial withdrawal at 0 hpw and a second withdrawal at the assigned post-withdrawal time point), two paired statistical analyses were performed. The first compared the total number of gated hemocytes (defined as the total number of events within the two gated regions, GI-like + GII-like) between the two sampling times, whereas the second compared the percentage of GI-like hemocytes over the total number of gated hemocytes between the same paired samples. Shapiro–Wilk and F tests were first applied to assess for normality and homoscedasticity, if (*p* > 0.05) the data were analyzed using parametric paired t-test, while if (*p* < 0.05) the Wilcoxon signed rank test was applied, using PAST software [78]. Statistical significance was set at α = 0.01. The rationale for applying a more stringent significance threshold to the flow cytometry analyses is that these experiments relied on paired comparisons, in which inter-individual variability was substantially reduced by directly comparing each individual with its own baseline condition. Under these circumstances, we adopted a more conservative significance level to minimize the risk of false-positive findings. To complement the paired analyses, a second, non-paired model-based time-series analysis was performed to characterize the overall temporal dynamics of the total number of gated hemocytes and the percentage of GI-like hemocytes using the paleoTS package implemented in R v4.5.1. This statistical approach has been widely applied to describe evolutionary patterns in paleontological ordered time series [59,60,61,62,63], and here it was used to capture the global temporal dynamics of hemolymph repopulation. For each time point [0 hpw (all 84 animals), followed by 1.5, 3, 6, 9, 18, 24, and 48 hpw (12 animals per time point)], mean values, variances, and the number of replicates were calculated and used to construct paleoTS objects. Four alternative evolutionary models were fitted: (i) Stasis, where the variation within individual time intervals is not significantly larger than the variance of the entire time series [60,62]; (ii) Unbiased Random Walk (URW), describing successive changes that lack a consistent directional trend [61,62]; (iii) General Random Walk (GRW), describing a directional shift in both mean and variance within each step of the temporal series [62]; and (iv) Ornstein–Uhlenbeck (OU), which is a particular case of directional change toward an adaptive optimum [62,63]. Model support was assessed by comparing corrected Akaike Information Criterion (AICc) values, which measure the relative quality of a statistical model for a given set of data [79]. The AIC statistic balances how well a model fits the data with its complexity, and the model with the lowest AIC value is preferred [63].

### 4.4. Hemocyte Slide Preparation

Morphological analysis of hemocytes was conducted on 3 snails, and hemolymph was collected as described in Section 4.2, with an initial withdrawal and a second sampling 9 hpw. Hemocyte slides were obtained by cytocentrifugation using Cytospin II (Shandon Instruments, Runcorn, UK). Immediately after hemolymph withdrawal, 2 samples of 150 μL per animal were centrifuged at 400 rpm for 3 min. The spots were fixed and stained using the BIO-DIFF^®^ kit (BioGnost, Zagreb, Croatia), air dried, and mounted with Eukitt^®^. Images were taken with a Nikon Eclipse Ni-E microscope (Leica Microsystems Srl, Milan, Italy) at the National Biodiversity Future Centre (NBFC) microscopy lab of the University of Modena and Reggio Emilia, Modena, Italy.

### 4.5. Dissection and Sampling of PK

PKs were collected from 19 snails for histological analysis. Four untreated animals served as controls, whereas 3 animals were sacrificed at each of the following time points after hemolymph withdrawal: 1.5, 6, 18, 24, and 48 hpw. Dissections were performed following cold anesthesia by placing the animals on granular ice for 30 min. After cracking the shell, PKs were immediately removed using dissection scissors and forceps. These samples were fixed for morphological analyses. For the gene expression analysis, PKs were collected from 18 snails: 6 controls, 6 at 18 hpw and 6 at 24 hpw. These samples were immediately processed for RNA extraction to be used in RT-qPCR experiments.

### 4.6. Slide Preparation and Staining, Image Analysis, and Statistical Analysis Histological Data

Samples dissected for the histological analysis were fixed in freshly prepared Bouin’s solution (15 mL saturated solution of picric acid, 5 mL formalin, 1 mL acetic acid) for approximately 6 h RT. Fixed tissue samples were stored in 70% ethanol at 4 °C until processing. Samples in clear ethanol 70% underwent dehydration through an ascending scale of graded ethanol series, clarification with xylene, and embedding in Paraplast^®^ (Sigma-Aldrich, St. Louis, MO, USA). The Paraplast^®^-embedded tissues were sectioned transversely into 7 µm-thick sections, obtaining 4–5 slides per sample with 30 sections each. Before staining, slides underwent standard rehydration through a descending ethanol series, as described by Ferri et al. [80]. One slide per sample was processed for staining with Mayer’s Hematoxylin–Eosin (HE) (Merk, Darmstadt, Germany) and mounted with Eukitt^®^ (Merk, Darmstadt, Germany), while two slides were stained with DAPI (1 µg/mL, 10 min) and mounted with Mowiol^®^ (Merk, Darmstadt, Germany).

Images from histology were acquired with Nikon Eclipse Ni-E microscope (Leica Microsystems Srl, Milan, Italy). For each DAPI-stained slide, images were taken by the operator from 4 different sections using a 4× objective. Images were then analyzed using “scanR” analysis software (v. 3.0.1, Olympus, Tokyo, Japan) (Inter-departmental Center of Big Instruments, C.I.G.S., University of Modena and Reggio Emilia), which was used to identify hemocyte islets within the renal parenchyma by manually applying an intensity threshold and a minimum object size criterion. In more detail, images belonging to the same slide were acquired under identical acquisition settings and analyzed as a single image set. For each image set, the operator manually defined the minimum object size and signal intensity threshold required to exclude the surrounding parenchyma and selectively identify the hemocyte islets (Figure S4). The adequacy of the segmentation parameters for the identification of the hemocyte islets was verified by visual inspection. Once established, the same parameters were applied uniformly and the accuracy of segmentation was visually checked by the operator for all images within the corresponding set. The area of all the hemocyte islets (A_hi_) was then calculated by “scanR” and related to the area of the section (A_tot_) to obtain a hemocyte islet area value, calculated as A_hi_/A_tot_ ratio.

A_hi_/A_tot_ differences between controls and experimental groups were assessed applying Dunnett’s test, (*p* < 0.05 set for statistical significance) using DescTools packages implemented in R (v.4.5.1). To characterize the overall temporal dynamics of A_hi_/A_tot_, the complementary model-based time-series analysis was subsequently performed using the paleoTS package, as described above.

### 4.7. Whole Transcriptome Expression Profiling and Sequence Analysis

To obtain preliminary in silico expression data of *Pc*TGase (Gene ID: LOC112576690) and *Pc*Hhex (Gene ID: LOC112564124), transcript abundance values expressed as transcripts per million (TPM) were retrieved from 152 tissue/organ-specific publicly available RNA-seq datasets deposited in the NCBI Sequence Read Archive (SRA) as described by Ferri et al. [32]. More specifically, we focused our analysis on the 134 tissue/organ-specific transcriptomes with more than three available replicates (Supplementary Table S2). Detailed information on the expression datasets is reported in the metadata table retrievable from Ferri et al. [32].

The expression values related to the *Pc*TGase and *Pc*Hhex genes were extracted and plotted by tissue/organ using tidyverse and ggplot packages implemented in R v.4.5.1. Structural domains of *Pc*TGase and *Pc*Hhex were predicted from the protein sequences using SMART, including Pfam domains database in the analysis [56].

### 4.8. FISH Protocol

FISH experiments were conducted on PK samples collected from 3 individual snails and fixed overnight at 4 °C in 4% paraformaldehyde in 1× PBS. The dehydrated samples were embedded in Paraplast^®^ after an intermediate infiltration with xylene [32]. Sections of 10 µm deposited on charged slides were processed for FISH using riboprobes for *Pc*TGase and *Pc*Hhex kindly provided by the Sánchez Lab (Stowers Institute for Medical Research, Kansas City, MO, USA). The FISH protocol was applied on rehydrated tissue sections from the PK of control animals, as described by Montanari et al., 2020 [22]. For each animal, a minimum of 10 slides were prepared, each carrying 10 sections. Nuclei were counterstained with DAPI (1 µg/mL) incorporated directly into Mowiol^®^ mounting medium. Images were acquired with a Leica TCS SP8 confocal microscope (Leica Microsystems Srl, Milan, Italy) at the C.I.G.S. (University of Modena and Reggio Emilia, Modena, Italy).

### 4.9. RNA Extraction and RT-PCR

Total RNA was purified using the Quick-RNA™ Miniprep Kit (Zymo Research, Irvine, CA, USA) according to the manufacturer’s protocol. RNA purity and concentration were assessed with a NanoDrop™ ND-1000 spectrophotometer (Thermo Fisher Scientific, Waltham, MA, USA). Approximately 1 µg of total RNA from each sample was reverse transcribed into cDNA using the iScript™ cDNA Synthesis Kit (Bio-Rad Laboratories, Hercules, CA, USA) following the manufacturer’s instructions. The resulting cDNA (1 µL) served as a template for RT-PCR amplification using GoTaq^®^ DNA Polymerase (Promega, Madison, WI, USA). Each reaction (25 µL final volume) contained the following: 1× GoTaq^®^ Reaction Buffer, 0.2 mM of each dNTP, 0.5 µM of forward and reverse primers for each gene (see Supplementary Table S1), and 1.25 U of GoTaq^®^ DNA polymerase (0.125 µL per reaction). The thermal cycling program was as follows: initial denaturation at 94 °C for 2 min; 35 cycles of denaturation at 94 °C for 30 s, annealing at 60 °C for 30 s, and extension at 72 °C for 1 min; followed by a final extension at 72 °C for 5 min. Amplicons were resolved by electrophoresis on 1.5% agarose gels stained with ethidium bromide and visualized under UV light.

### 4.10. RT-qPCR and Statistical Analysis

For gene expression analysis, 6 samples from the control group, 6 samples collected at 18 hpw, and 6 samples at 24 hpw (Section 4.5) underwent RT-qPCR. Quantitative PCR (qPCR) was performed on the synthesized cDNA (Section 4.9) using the SsoAdvanced™ Universal SYBR^®^ Green Supermix (Bio-Rad Laboratories, Hercules, CA, USA) in combination with specific primers for the reference gene ribosomal protein L5 (*Pc*RpL5, XM_025256997.1) and each of the target genes *Pc*TGase (XM_025259333.1) and *Pc*Hhex (XM_025238728.1) (Supplementary Table S1). Reactions were prepared following the manufacturer’s instructions. Thermal cycling was conducted under the following conditions: initial denaturation at 95 °C for 2 min, followed by 40 cycles of denaturation at 95 °C for 10 sec and combined annealing/extension at 60 °C for 30 sec. All reactions were run in triplicate using a CFXDuet Real-Time PCR Detection System (Bio-Rad Laboratories) at the NBFC molecular biology laboratory (University of Modena and Reggio Emilia, Modena, Italy). At the end of each run, a melting curve analysis was performed to confirm amplification specificity. Relative gene expression levels were calculated using the 2^−ΔΔCt^ method [81], with the control group serving as reference. Data normality and variance homogeneity were verified using Levene’s test for homoscedasticity (*p* > 0.05). Differences among experimental groups were assessed by one-way ANOVA, using PAST statistical software [78]. Statistical significance was set at *p* < 0.05.

## Figures and Tables

**Figure 1:**
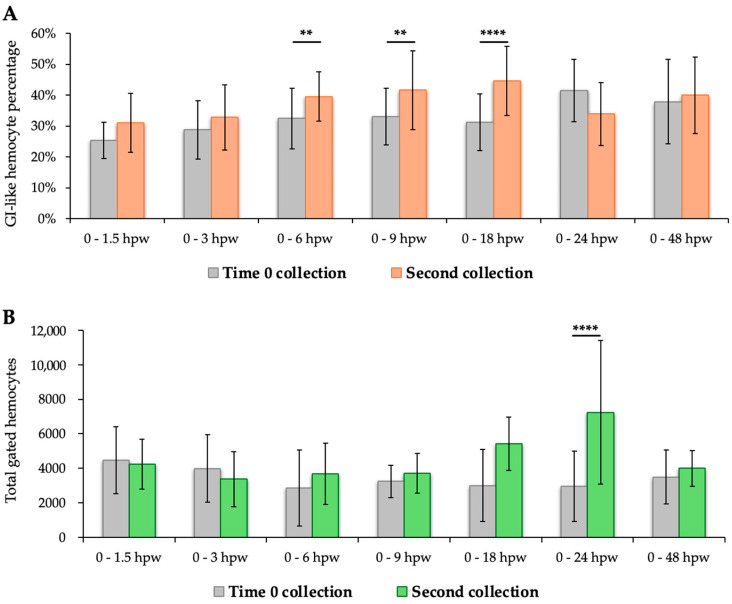
Flow cytometry analysis of GI-like hemocyte percentage (**A**) and total gated hemocytes (**B**) at time 0 (gray) and after n hours post-withdrawal (hpw), applying two-sample paired *t*-test and Wilcoxon test between second and first hemolymph withdrawal (α = 0.01). GI hemocyte percentage increased significantly after 6, 9 (** *p* < 0.01), and 18 hpw (**** *p* < 0.0001, two-sample paired *t*-test), while total number of gated hemocytes increased significantly after 24 hpw (**** *p* < 0.0001, two-sample paired *t*-test).

**Figure 2:**
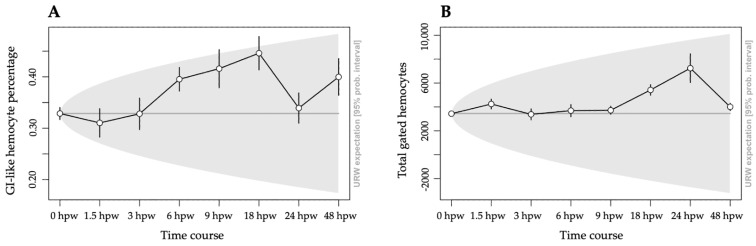
Model-based temporal series analysis of circulating hemocytes within 48 hpw. (**A**) Model-based temporal series analysis of GI-like hemocyte percentage. URW Akaike weight = 0.688, Stasis Akaike weight = 0.188. dAICc = 2.597. (**B**) Model-based temporal series analysis of total gated hemocytes. URW Akaike weight = 0.623, Stasis Akaike weight = 0.335. dAICc = 1.241. For both time series, the URW model was the best-fitting model.

**Figure 3:**
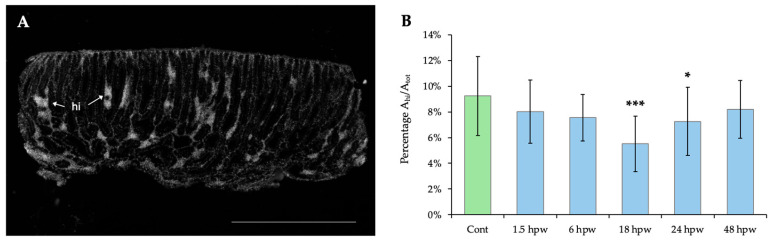
Identification of *P. canaliculata* PK hemocyte islets and quantification of A_hi_/A_tot_ ratio following hemolymph withdrawal. (**A**) Representative image of a PK section stained with DAPI used to calculate the A_hi_/A_tot_ ratio. Scale bar = 1 mm. Abbreviation: hi = hemocyte islet. (**B**) Changes in the A_hi_/A_tot_ ratio following hemolymph withdrawal. The A_hi_/A_tot_ ratio decreased significantly at 18 hpw and 24 hpw compared to controls (green), according to Dunnett’s post hoc test (α = 0.05 was set for statistical significance; * *p* < 0.05; *** *p* < 0.001).

**Figure 4:**
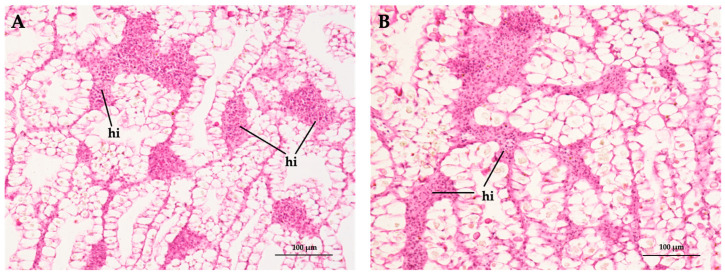
Histology of control PK (**A**) and PK at 18 hpw (**B**). One representative animal from a group of three is reported. Hemocyte islets are indicated. No differences in the morphology or histological organization of renal hemocyte islets were detected. Scale bar = 100 μm. Abbreviation: hi = hemocyte islet.

**Figure 5:**
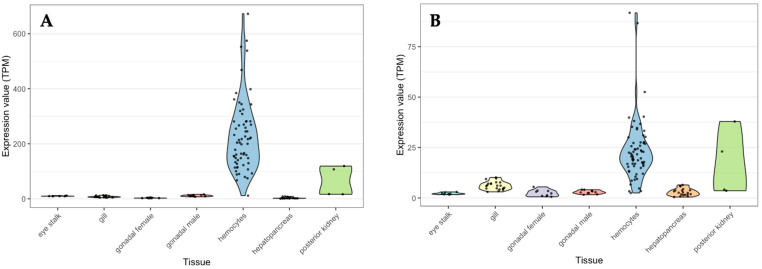
Expression values of *Pc*TGase (LOC112576690, (**A**)) and *Pc*Hhex (LOC112564124, (**B**)). Violin plots show the distribution of the expression values reported as transcripts per million (TPM) across a selection of tissues/organs with at least three biological replicates. Hemolymph (i.e., circulating hemocytes) showed the highest average expression of both *Pc*TGase and *Pc*Hhex, followed by PK.

**Figure 6:**
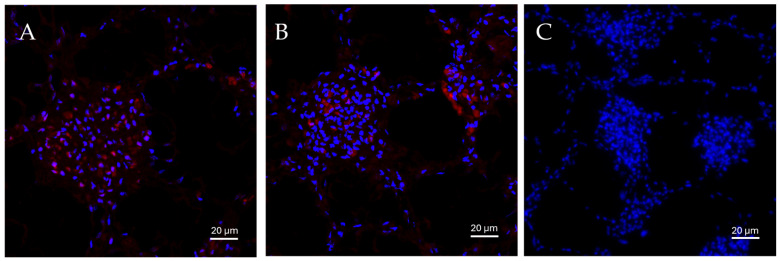
FISH images showing the localization of *Pc*TGase and *Pc*Hhex expression in control PK. Both the target molecules were detected within hemocyte islets. Cells expressing *Pc*TGase (**A**) or *Pc*Hhex (**B**) are shown in red. Nuclei were counterstained with DAPI (blue). Negative control (no probe) (**C**).

**Figure 7:**
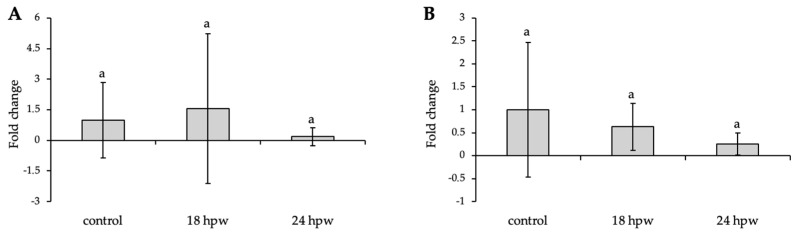
RT-qPCR analysis of *Pc*TGase (**A**) and *Pc*Hhex (**B**) expressions in control PKs and in PKs collected at 18 and 24 hpw. No significant differences were detected among groups in any condition (*p* > 0.05).

## Data Availability

The processed data supporting the findings of this study are provided in the article and its Supplementary Material. The publicly available RNA-seq datasets analyzed in this study are available from the NCBI Sequence Read Archive (SRA), and the corresponding SRA accession numbers are reported in the metadata in Ferri et al., 2025 [32].

## Supplementary Materials

The following supporting information can be downloaded at: https://www.mdpi.com/article/10.3390/ijms27156619/s1.
