# Supplementary material for: Parallel Recovery Dynamics of Circulating and Tissue-Resident Hemocytes Following Hemolymph Withdrawal in Pomacea canaliculata
Source: Int J Mol Sci. 2026 Jul 24;27(15):6619. doi: 10.3390/ijms27156619 (PMC13467024; doi:10.3390/ijms27156619)
Supplement: Supplementary file 1 [file ijms-27-06619-s001.zip › ijms-4377463-supplementary.pdf]

Supplementary Materials

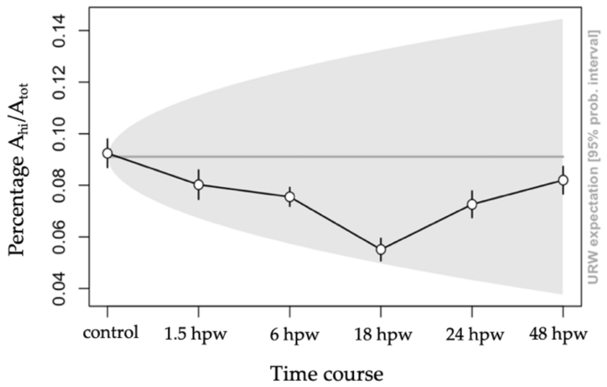

Figure S1. Model-based temporal series analysis of hemocyte islet area (A_{hi}/A_{tot}) within 48 hours after hemolymph withdrawal (hpw). Temporal series is represented by URW as the best-fitting model.
URW Akaike weight = 0.609, Stasis Akaike weight = 0.387. dAICc = 0.908

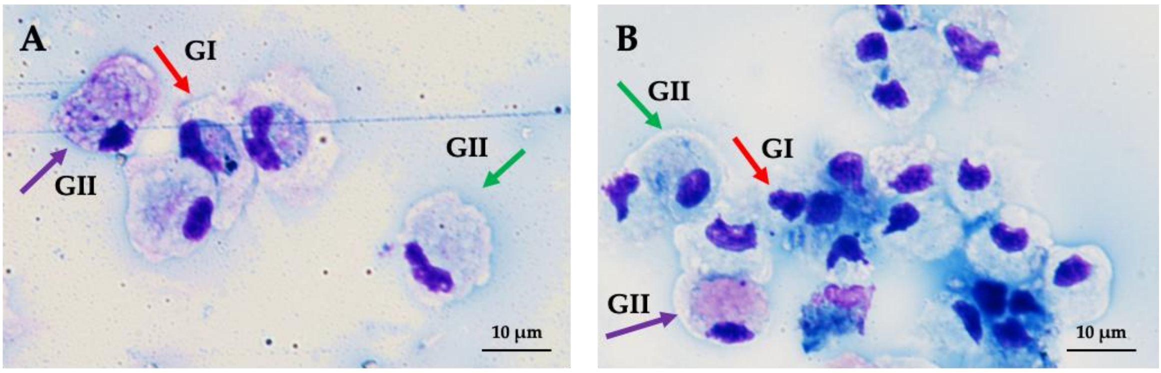

Figure S2. *P. canaliculata* circulating hemocytes after centrifugation and BIO-DIFF® (BioGnost) staining. Collection time 0 hpw (**A**) and 9 hpw (**B**). One animal representative of a group of 3 is reported. Nuclear morphologies from cells in active mitosis were undetectable in control slides (time 0 hpw) and during hemolymph repopulation (time 9 hpw).
Scale bar = 10 µm. Abbreviation: GI = Group I hemocytes; GII = Group II hemocytes.
Red arrows = Group I hemocytes; Green arrows = Group II agranular hemocytes; Purple arrows = Group II granular hemocytes

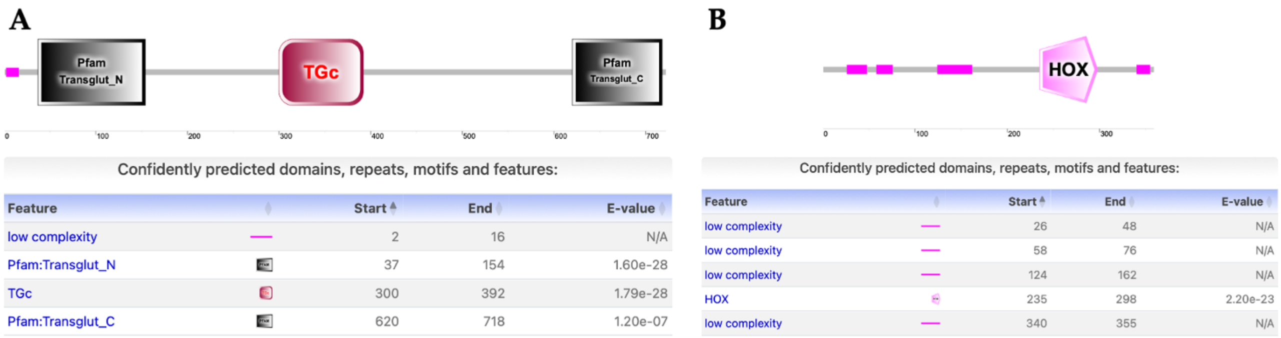

Figure S3. Conserved domain architecture of candidate hematopoietic genes from *P. canaliculata* identified using SMART
A) Gene ID LOC112576690 (XP_025115118.1) contains three conserved domains characteristic of transglutaminase proteins (Transglut_N, Tgc, Transglut_C).
B) Gene ID LOC112564124 (XP_025094513.1) shows a conserved HOX domain consistent with the similarity to the Hhex transcription factor.

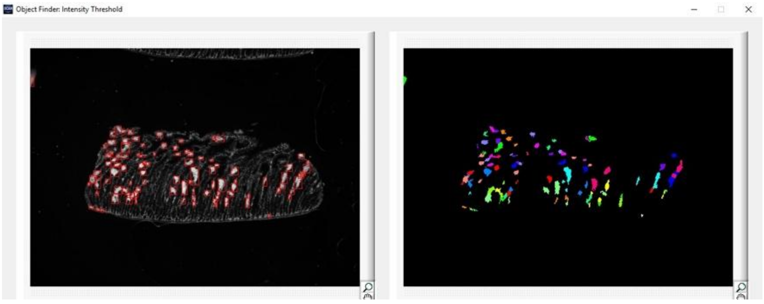

Figure S4. Screenshot of scanR-mediated identification of hemocyte islets. On the left the first identification provided by the software. On the right, the results of segmentation after the manual validation of the areas.

Table S1. List of the primers used RT-qPCR experiments

	Gene	Primer sequence
**RT-qPCR**	*Pc*RpL5 (XM_025256997.1)	F: 5′-CGTATGCCAGAATTGAGGGT-3′<br>R: 5′-CAACATCCAAGTATGCACGG-3′
	*Pc*TGase (XM_025259333.1)	F: 5′-AGTCGCAATGTGAACCGCCA-3′<br>R: 5′-TGAGCACGATGCTGTCGTCG-3′
	*Pc*HHex (XM_025238728.1)	F: 5′-ACCATTTCTTCAGCGACCAC-3′<br>R: 5′-TCCATTTTGCCCTTCTGTTC-3′
